# PALLIATIVE AND END-OF-LIFE CARE INTERVENTIONS FOR INDIVIDUALS EXPERIENCING HOMELESSNESS: A SCOPING REVIEW

**DOI:** 10.1093/geroni/igac059.2511

**Published:** 2022-12-20

**Authors:** Pilar Ingle

**Affiliations:** University of Denver, Lakewood, Colorado, United States

## Abstract

Despite the benefits of palliative and end-of-life (PEOL) care for individuals with life-limiting illness, barriers to accessing this care persist among older adults experiencing homelessness. A number of studies have identified barriers to PEOL care from the perspectives of unhoused individuals and other relevant stakeholders; however, few empirically studied interventions exist to support evidence-based guidelines for PEOL care for this population. This scoping review aimed to identify PEOL interventions for adults experiencing homelessness. The search was conducted in May 2021 and yielded 480 results, from which 50 full-text articles were closely reviewed for inclusion. Sources were included if they reported findings of original, empirical research or quality improvement projects evaluating PEOL services tailored for individuals experiencing homelessness. A total of 13 articles representing 12 studies published between 2006-2021 were included in the review. Over half (n = 7) of the studies described interventions to promote advance care planning documentation, five described interventions embedding PEOL services within homeless shelters, and one described a community outreach program to increase access to PEOL services. Nearly all studies reported positive outcomes on participant perspectives, advanced directive completion rates, or cost analysis. None of the studies included clinical palliative care outcomes. Although most studies reported promising results, service and healthcare providers expressed concerns about continuing challenges and longevity of the programs. The body of literature encompassing PEOL interventions for people experiencing homelessness is limited but growing. Future research may benefit from the inclusion of clinical outcomes and efforts to reduce structural barriers to providing care.

